# Legume Proteins in Food Products: Extraction Techniques, Functional Properties, and Current Challenges

**DOI:** 10.3390/foods14091626

**Published:** 2025-05-04

**Authors:** Grazielle Náthia-Neves, Adane Tilahun Getachew, Ádina L. Santana, Charlotte Jacobsen

**Affiliations:** 1 National Food Institute, Technical University of Denmark, 2800 Lyngby, Denmark; atige@food.dtu.dk (A.T.G.); chja@food.dtu.dk (C.J.); 2 Grain Science and Industry Department, Kansas State University, Manhattan, KS 66506, USA; adina@ksu.edu

**Keywords:** protein extraction, emerging techniques, green extraction methods, legumes, plant-based proteins

## Abstract

The aim of this review is to provide a comprehensive overview of protein extraction from legume sources, with a focus on both conventional and emerging techniques. Particular attention is given to the impact of innovative methods on protein functionality, a key factor for food applications. Due to their nutritional profile and techno-functional properties, legumes are increasingly regarded as promising alternatives to animal-based protein sources in the food industry. Traditional extraction methods, such as alkaline and acidic extraction, are discussed and compared with novel approaches including enzymatic extraction, ultrasound-assisted extraction (UAE), microwave-assisted extraction (MAE), ohmic heating (OH), subcritical water extraction (SWE), deep eutectic solvents (DES), and dry fractionation. The potential of these emerging technologies to improve protein yield and functionality is critically assessed, alongside key challenges such as scalability, cost-effectiveness, and potential allergenicity. This review also identifies current research gaps and highlights opportunities for innovation in sustainable protein extraction. Therefore, this review contributes to the development of more efficient, functional, and sustainable protein ingredients production, highlighting the role of innovative extraction technologies in shaping the future of plant-based foods.

## 1. Introduction

The growing interest in plant-based proteins is driven by the urgent need to reduce the consumption of animal-based proteins, which are significant contributors to greenhouse gas emissions. Furthermore, plant-based proteins are more cost-effective than animal-derived sources, making them accessible to a wider range of groups. They also play a vital role in supporting vegan and vegetarian diets.

Legumes, a key source of dietary protein, serve as a primary protein supply in many regions of the world [1]. Belonging to the *Fabaceae* family, legumes represent the second-largest group of seed plants, encompassing around 600 genera and approximately 13,000 species [2]. Among these, about 60 species have been domesticated, including soybeans (*Glycine max*), mung beans (*Vigna radiata* L.), lupins (*Lupinus* spp.), chickpeas (*Cicer arietinum* L.), and lentils (*Lens culinaris*) [3]. The term ‘legume’ derives from the Latin word ‘legumin’, meaning the harvesting of grains within pods, while ‘pulses’, another term for legumes, originates from the Latin word ‘puls’, referring to a porridge made from ground grains [2,3].

Storage proteins in legumes are categorized according to Osborne’s classification based on their solubility: albumins are soluble in water, globulins in saline solutions, prolamins in ethanol-water solutions, and glutelins in acid/alkali solutions [4]. Among legumes, globulins are the most abundant class of storage proteins. From a nutritional perspective, legume proteins are relatively low in sulfur-containing amino acids (e.g., methionine, cysteine, and tryptophan) but rich in lysine and leucine, two essential amino acids often limited in cereal grains [1]. Thus, legume and cereal proteins are nutritionally complementary in their amino acid profiles [1].

**Figure 1:**
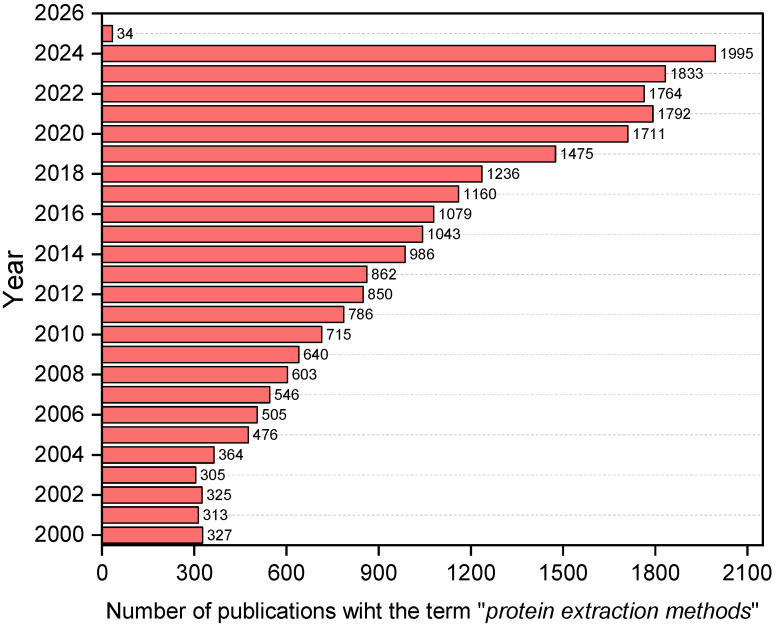
Number of publications using the term “protein extraction methods” in the Web of Science™ database (2000–2025). Search conducted on 27 January 2025.

The versatility of legume proteins, widely used as foaming agents, emulsifiers, stabilizers, and as nutritional ingredients [5], has significantly increased interest among researchers. This is evidenced by the growing number of scientific publications on the “protein extraction methods” topic in research articles, reviews, and book chapters indexed in the Web of Science™ database from 2000 to 2025 (Figure 1). This bibliometric analysis was used to illustrate the increasing scientific attention toward the subject; however, the references cited in this review are not limited to this timeframe.

Traditionally, protein extraction has been performed using conventional methods, often relying on chemical reagents and prolonged extraction times. For instance, most studies use non-food-grade solvents during protein extraction. Hexane is commonly employed for defatting raw materials, while water, with pH adjustments using chemicals like NaOH and HCl, is predominantly used for extraction and precipitation, respectively [6]. However, as food ingredients, proteins must be free from hazardous substances, making purification steps more complex, costly, and time-consuming.

**Figure 2:**
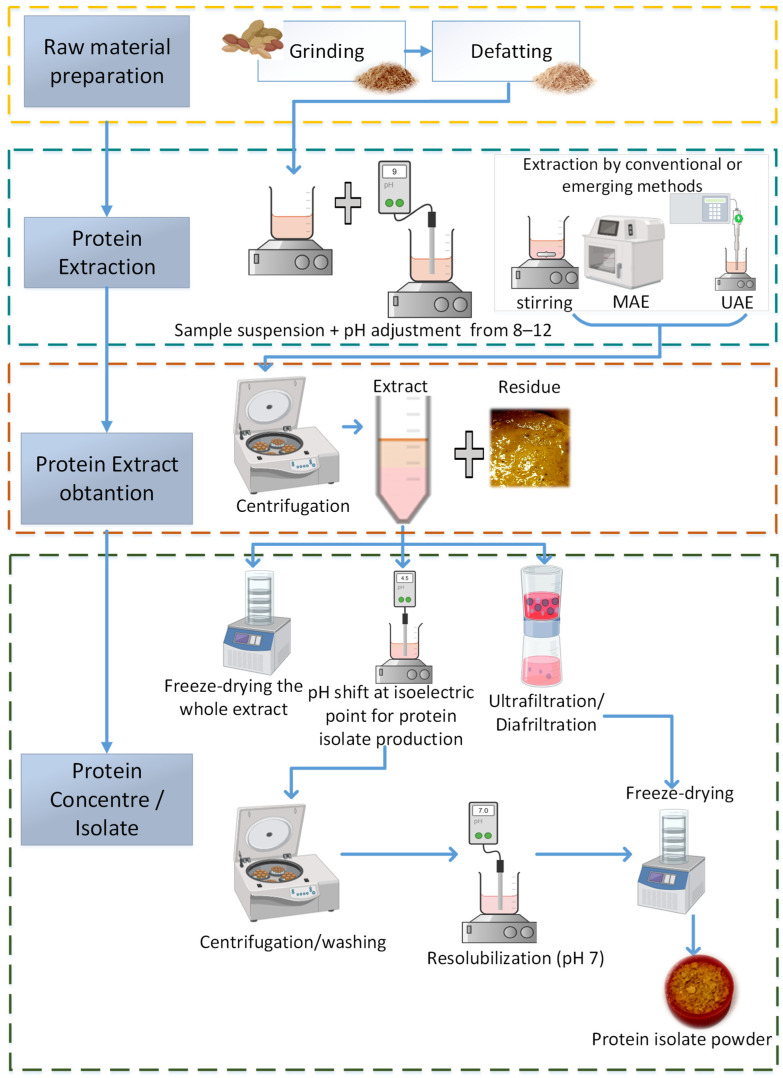
Schematic representation of the common steps used for obtaining proteins from plant sources.

Plant proteins are usually extracted, isolated, and purified before they are used as food ingredients. The common steps for obtaining proteins from plants are shown in Figure 2 and include (i) raw material preparation (e.g., cleaning, grinding, and other pretreatments); (ii) oil removal; (iii) protein extraction using water or an alkaline medium; (iv) protein precipitation at the isoelectric point; and (v) protein purification. Pretreatment techniques applied during raw material preparation (step i) can significantly affect protein extractability, yield, and functional performance. For example, milling or fine grinding increases surface area and enhances mass transfer during extraction. Defatting is a crucial step especially for oil-rich legumes, as it prevents lipid–protein interactions that hinder extraction and can improve protein purity [7]. Soaking facilitates the softening of seed structure and improves extractability; blanching can inactivate antinutritional factors and enhance protein solubility; and dehulling or dehooking, which removes the seed coat, reduces fiber interference during extraction [8,9]. Additionally, solid-state fermentation has been explored as a biological pretreatment that can hydrolyze storage proteins and modify their functional properties [9]. Each of these pretreatments can significantly affect not only the efficiency of protein recovery but also the physicochemical and functional properties of the extracted proteins.

Recently, there has been a growing trend toward using green technologies in all steps of protein extraction and isolation processes. For example, researchers such as Náthia-Neves et al. [7] and Lazdiņa et al. [10] have proposed the use of supercritical CO_2_ to remove oil from lupin (*Lupinus angustifolius* L.) and Japanese quince (*Chaenomeles japonica*), respectively, before protein extraction. Additionally, alternative solvents such as sodium carbonate for extraction and organic acids like citric and lactic acids for precipitation have been explored [11,12]. Moreover, advancements in emerging technologies, such as ultrasound-assisted extraction (UAE) and microwave-assisted extraction (MAE), have demonstrated the potential to promote green solvents, reduce energy consumption, and minimize solvent use, aligning with sustainable food processing goals [13]. These methods may also be well-received by environmentally conscious consumers. Beyond enhancing protein yield, these technologies can induce changes in the secondary and tertiary structures of proteins, thereby altering their functional properties [13]. These structural modifications can make the extracted proteins suitable for diverse applications in the food industry, and thereby further expand their utility.

Some reviews discuss protein extraction from legumes, but few provide an integrated perspective on how extraction processes affect protein functionality, a crucial factor for food applications. In this sense, this review aims to serve as a reference for researchers and food industry professionals by providing a comprehensive overview of recent advances in legume protein extraction methods, with particular attention to their effects on protein functionality. The main emphasis is placed on non-conventional techniques, which have shown greater potential for achieving high protein yield and quality. By integrating data on protein content, extraction processes, and protein functionality, this work contributes to expanding the available knowledge in this evolving field. Furthermore, it discusses the current opportunities and challenges, ultimately outlining future directions for research and innovation.

Given the broad range of extraction methods and their influence on protein properties, a structured search was conducted to evaluate the current state of research in this area. The literature was collected through a systematic search in the Web of Science™ and Scopus databases. The main keywords used included combinations of “protein extraction”, “legume proteins”, “pulse proteins”, “functional properties”, “bioactivity”, “extraction methods”, emerging technologies, and “processing techniques”. Most of the included works were published between 2000 and 2025; however, earlier studies were also considered when relevant for historical background or methodological development. The inclusion criteria comprised peer-reviewed articles, review papers, and book chapters focused on legume-derived proteins, their extraction techniques, and application in food systems. After title and abstract screening and the application of inclusion/exclusion criteria, a curated set of approximately 200 studies was used to support the discussion throughout this review article.

## 2. Legume Protein Sources—Functionality, Bioactivity, and Digestibility

Legumes are defined as plants belonging to the *Fabaceae* family, including their leaves, seeds, and pods. Legume proteins are increasingly recognized as key ingredients in the development of plant-based and functional foods due to their high nutritional value, functional versatility, and environmental sustainability [4]. They are rich in essential amino acids (particularly lysine and leucine), essential B vitamins, essential minerals, dietary fiber, and bioactive compounds, making them attractive for both improving nutritional profiles and delivering health benefits, such as cholesterol reduction, glycemic control, and antioxidant activity [2,14]. From a technological aspect, legume proteins exhibit desirable functional properties including emulsification, gelation, foaming, and water-holding capacity, which are essential for the formulation of a wide range of food products such as meat and dairy alternatives, protein-enriched beverages, and bakery goods [14]. Moreover, legumes have a low environmental footprint, requiring less water and nitrogen fertilizer than many animal-based protein sources [15]. This aligns with the growing consumer demand for sustainable and ethical food choices, driving their increasing inclusion in plant-based diets and health-promoting formulations. Consequently, the food industry is increasingly favoring legume-derived proteins as a viable, functional, and sustainable alternative to animal proteins. Proteins obtained from soybeans (*Glycine max*) and peas (*Pisum sativum* L.) are the most commercialized. Other conventional legume sources include peanuts (*Arachis hypogaea*), lentils (*Lens culinaris*), and lupins (*Lupinus* spp.—considered conventional depending on the region), and various types of beans. Soybeans (*Glycine max* L. Merr.) are extensively utilized in food systems for their high protein content and technological versatility, with broad application in meat and dairy analogs, as well as in sauces [16]. However, soybean proteins are also recognized as common food allergens, with allergenic components including Gly m 5 (β-conglycinin) and Gly m 6 (glycinin), the major storage proteins [17]. These allergens can trigger IgE-mediated reactions, particularly in children, although many individuals outgrow the allergy with age [18]. Despite these concerns, soybeans remain highly popular in food applications due to their well-balanced amino acid profile, cost-effectiveness, and technological versatility [16]. Pea is another legume source with increasing interest in the food industry because of its low allergenicity and nutritional value [19]. Lupins have recently gained attention as a promising source of protein, and their use as food ingredients in the industry is steadily increasing [7]. They are incorporated into bakery products such as bread, biscuits, and pasta, enhancing the nutritional value by reducing refined carbohydrates and significantly increasing protein and dietary fiber content [20]. Lupins have also been processed into plant-based milk alternatives and used in the production of ice cream [20]. Beyond conventional sources, other legumes have been explored as alternative protein sources, including pigeon pea (*Cajanus cajan*) [21], jack bean (*Canavalia ensiformis*) [22], Acacia (*Acacia* spp.) [23], carob (*Ceratonia siliqua*) [24], faba bean (*Vicia faba*) [25], and hyacinth bean (*Lablab purpureus*) [26]. The protein content of the most common legumes is shown in Table 1. Legume proteins are mainly composed of globulins (35–80%) and albumins (2–37%) [27]. Legumin (11S) and vicilin (7S) are the major globulins, whereas enzymes, enzyme inhibitors, and lectins belong to albumins [27]. Albumins are rich in lysine and sulfur-containing amino acids, while globulins have higher levels of aspartic and glutamic acids, resulting in distinct overall amino acid profiles [27]. For a more comprehensive understanding of the complete protein profiles of different legume sources, readers are referred to the following studies [27,28,29,30]. Non-conventional legume sources such as carob, hyacinth bean, and acacia are indigenous to developing nations, but their economic impact on international markets is not as intense in comparison to conventional ones, such as soybeans, peas, or lentils. Despite their superior dietary potential, they are commonly used as animal feed and for other agricultural purposes by resource-constrained farmers. The utilization of non-conventional legumes supports farmers to increase their production, crop diversity, and food security [31]. Proteins isolated from non-conventional sources have been considered of good quality compared to conventional sources. For instance, in acacia seed protein isolates, their amino acid composition and protein solubility were found to be higher than soy protein [32]. Despite their high protein content (up to 90%), faba beans have traditionally been used as animal feed [25]. Recently, however, their proteins have attracted growing interest for the development of meat analogs [33]. Carob is another underutilized protein source and there is a scarcity of current research on its protein attributes. Bengoechea et al. reported that the protein content detected from a commercial protein isolate of carob was 89.9% [24]. In the African locust bean tree (*Parkia biglobosa* (Jacq.)), 27% protein was found [34]. Lima bean (*Phaseolus lunatus* Linn) protein is another underused source that demonstrated high potential to stabilize emulsions in a recent study [35].

### 2.1. Functional Properties

The functional properties of proteins refer to their physicochemical characteristics that influence the behavior of proteins during food processing, storage, and consumption. These properties play a crucial role in determining the quality, texture, stability, and sensory attributes of food products. Key functional properties include solubility, water- and oil-holding capacities, emulsifying and foaming abilities, gelation, and texturizing behavior. In addition, nutritional aspects such as amino acid profile and digestibility are also considered integral to protein functionality, particularly in the context of developing high-quality plant-based foods. In the following sections, each of these functional properties will be briefly discussed, along with their relevance in food formulation.

#### 2.1.1. Solubility

Protein solubility serves as a foundational characteristic that influences a wide range of functional attributes, making it a critical parameter in food formulation and processing. This property influences other functional attributes such as gelling and emulsion properties, as well as water and oil absorption capacities [46]. For instance, water solubility of protein affects the appearance and texture of food products. Moreover, the type of process, the pH, and type of solvent considerably affect the solubility of protein. For instance, peanut protein isolates obtained via alkali extraction showed water solubility of 83.61%, which was higher than the 26.55–27.79% observed for protein extracted via ultrasound (US) and microwave (MW) [44]. Acacia protein isolate showed a minimum solubility at pH = 4 and maximum solubility at pH = 12 [47]. Similarly, pea protein isolates showed minimum solubility at pH = 4–5, and highest solubility at pH = 8–10 [48]. Likewise, the solubility of lima bean (*Phaseolus lunatus*) seed coats was lowest at a pH of 4, which is close to their isoelectric point, whereas it increased to 60–80% at pH 10 [49]. With respect to the effect of the solvent, the solubility of carob protein isolates in aqueous dispersions ranged between 3.53% (pure water) and 45.6% (0.1 M sodium borate buffer containing 0.5% sodium dodecyl sulfate and 1% dithiotreitol DTT) [24].

#### 2.1.2. Water- and Oil-Holding Capacity

The ability of food materials to absorb water is determined by their association with the complex system of water, whereas a food’s ability to absorb oil is related to its ability to retain taste and texture [50]. Previous knowledge of the water- and oil-holding capacity of a protein helps to predict the effect of the addition of protein on the characteristics of food systems to prevent losses of water or oil during processing, and subsequently, to avoid undesirable textures and sensory properties. Proteins with high water- and oil-holding capacity are important in various food systems, including meat substitutes, dairy analogs, and baked products [25,32]. Compared to soy protein isolate, the water-holding capacity of acacia bean protein isolates (2.8–4.7%) was similar, while their oil-holding capacity (4.5%) was higher [32]. Water-holding capacity of faba bean protein isolate was 62.72% [51]. Oil-holding capacities next to 170% were observed for tamarind seed isolates treated with US [52].

#### 2.1.3. Emulsifying and Foaming Properties

The emulsifying activity and emulsion stability indicate the potential of protein to form emulsions in water- and oil-based systems. The emulsifying activity and emulsion stability of proteins depend on their ability to adsorb at the oil–water interface, reducing interfacial tension and stabilizing the dispersed phase. Proteins with amphiphilic properties, containing both hydrophilic and hydrophobic regions, function as conventional emulsifiers by unfolding at the interface and forming a stabilizing film [53]. Alternatively, insoluble proteins can act as Pickering particles, stabilizing emulsions through steric hindrance rather than interfacial tension reduction [54]. Singh et al. (2023) observed the emulsifying activity and emulsion stability of Manilla tamarind (*Pithecellobium dulce*) seed protein isolates of 56.4%, and 52.48%, respectively [50]. However, these parameters decreased after treatments with autoclaving and US [50]. The highest emulsifying capacity of ganxet bean (*Phaseolus vulgaris*, var. ganxet) protein isolate of next to 70% was found at pH = 8 compared to other pH ranges studied [42]. Emulsification capacities ranging between 193.7% and 243.7% were observed in pea protein isolates extracted via salt extraction–dialysis [19]. Liu et al. observed that lima bean isolated via aqueous-alkali extraction resulted in 84.14% solubility and high emulsifying activity (15.97 m^2^·g^−1^) [35]. Foaming stability consists of the potential of a protein to produce stable foams. Foaming capacity is the ability of a protein to rapidly unfold and form a cohesive layer around gas bubbles, which ensures a uniform distribution of fine air cells in food matrices, promoting smoothness and lightness [25,42]. This ability depends on factors such as protein structure, solubility, surface hydrophobicity, and environmental conditions like pH and ionic strength. Badjona et al. observed that the foaming capacity of faba bean protein isolated via alkaline extraction was 65.56%, while its foaming stability reached 100% [25]. The highest foaming capacity of 65% was observed for ganxet beans at the lowest pH value of 2 [42].

#### 2.1.4. Gelling Properties

Gelling properties of protein are characterized by both liquid- and solid-like properties [55]. The gelation abilities involve the formation of a three-dimensional network via protein–protein, protein–solvent interactions responsible for semisolid and viscoelastic characteristics [4,56]. The parameters observed to influence strongly the properties of gel forming include the type of protein (source), protein content, pH, ionic strength, and temperature [57]. Assessing the gelling properties and their modification after interaction with additives (water, sugar, salts, and fats) and processing helps to predict the texture of final products. High viscoelasticity, adhesiveness, and water retention are characteristic of proteins of high gelling properties or abilities. Compared to animal protein, the gelling properties of plant proteins are inferior, which supported research to improve the quality of protein gels to replace the ones of animal origin in food and non-food products [58]. Bengoechea et al. (2017) observed that the gelling properties of carob protein isolate exhibited high viscoelasticity when a 30% protein concentration was used [24]. Wang et al. observed that the addition of chickpea (*Cicer arietinum* L.) protein isolate at 8% in hairtail surimi gels resulted in stronger interaction with myosin protein in hairtail and consequently an enhancement in gel ability was found [59]. Gel formulations with bean protein isolate at 17% and neutral pH showed great gelation ability, which was considered appropriate to be used in meat analogs [60].

#### 2.1.5. Digestibility

The higher digestibility of protein in the human gut serves as an indicator of low amounts of anti-nutrients [26]. Anti-nutrients limit the utilization of protein by reducing protein digestibility, leading to pathogenesis when the raw material is not properly processed. Some anti-nutrients include trypsin inhibitors, phytates, divicines, saponins, and tannins [61]. Processing of legumes has been associated with improvement in the protein’s digestibility via deactivation of anti-nutritional factors and the subsequent release of proteins [62]. For instance, the digestibility of locust bean protein improved considerably (by 74.29%) after 72 h fermentation [63]. Adiamo et al. (2023) observed that roasting of acacia seed flour for 7 min significantly increased protein digestibility [23]. In a separate study, they found high protein digestibility in two varieties of acacia seed that ranged between 64.2 and 79.3%, which was lower compared to the 89.5% found for soy protein isolate [32]. Mohan and Mellem (2020) observed in vitro protein digestibility of 88.47% of water hyacinth bean protein isolated via isoelectric precipitation using the pH drop assay [26]. Additionally, peanut protein extracted via alkaline extraction showed in vitro digestibility of 95.16%, which was significantly higher than the digestibility found in protein extracted via ultrasound-assisted extraction [44].

#### 2.1.6. Bioactivity

There is a growing interest in the potential of using plant-based proteins as an alternative to animal protein for human consumption. Among the various plant-based proteins studied for their health benefits, soybean is the most widely researched source. For example, Tang et al. (2009) observed that the consumption of soy protein did not significantly increase postprandial myofibrillar protein synthesis in elderly patients, unlike whey protein, which resulted in an 18% higher synthesis [64]. This finding is important, as muscle protein synthesis plays a crucial role in maintaining skeletal muscle mass [65]. In another clinical trial, George et al. (2020) observed that the consumption of both soy and casein protein in individuals significantly reduced inflammation, with no significant difference between the treatments [66]. These results suggest that plant-based proteins may offer similar anti-inflammatory benefits to animal proteins. In vitro studies have also highlighted the bioactivity of plant-based proteins. For example, chickpea protein isolates extracted with carbohydrases and alkali demonstrated antioxidant potential, scavenging 50–60% of ABTS free radicals. This antioxidant activity is valuable in food applications as it can help extend the shelf life of products by delaying oxidation [67]. A key factor contributing to the health benefits of legume proteins is the presence of bioactive peptides, which are generated through protein hydrolysis [68]. These peptides have been shown to have various health-promoting properties. For instance, bioactive peptides derived from yellow pea protein have demonstrated neuroprotective effects, including antioxidant activity and the inhibition of enzymes associated with memory loss and cognitive decline [69]. However, one challenge in utilizing these bioactive peptides is ensuring their stability during gastrointestinal digestion to maximize their health benefits [70].

## 3. Methods to Obtain Legume Proteins

Protein-based products are classified according to the protein content and production method. According to the protein percentage, the products are classified as (a) flour (up to 65% protein), (b) concentrates (65–90%), and (c) isolates (higher than 90%) [8]. With respect to the production method, protein-enriched products are obtained via wet and dry processing (dry fractionation via air classification) [4]. The knowledge of effects of processing on the functionality of proteins or protein-rich food products is needed to improve the quality of products. Multiple methodologies have been studied to obtain proteins and enhance the functionality of legume proteins, including extraction using alkali, ultrasound (US), and microwave (MW). In the next subtopics, we introduce the recent findings on methods used to obtain protein from legumes.

### 3.1. Conventional Extraction Methods

Conventional extraction techniques use organic solvents, alkali, and acids [13]. Water or aqueous extraction of proteins is the simplest method to extract protein with hydrophilic characteristics. For instance, Liu et al. (2008) extracted albumins and globulin in two chickpea varieties with water and salt solutions [71]. In contrast, proteins with lipid-binding affinity, often due to hydrophobic regions, are more readily extracted using moderately polar to non-polar solvents such as propane and isopropanol. The proteins that bind with lipids are referred to as lipid transfer proteins (LTP). The LTP are low-molecular weight proteins containing a hydrophobic cavity that allows them to bind with lipids [72]. In peanuts, oleosins are LTPs that bind with lipid droplets enclosed by a monolayer of phospholipid [73]. Plankensteiner et al. (2011) observed that oleosins were highly extracted with the consecutive use of methanol, hexane, and ethanol [74]. The sources, extraction methods, yields, and protein content of products obtained by conventional techniques are summarized in Table 2.

#### 3.1.1. Water Extraction

Water extraction is one of the simplest and most environmentally friendly techniques for protein extraction. It typically involves the use of water as the sole solvent, with the extraction usually performed at the natural pH of the legume–water mixture (around pH 5–6), although some studies slightly adjust the pH to neutral (around pH 7) [75]. This method is particularly effective for extracting water-soluble proteins such as albumins [4]. Its main advantages include low cost, minimal chemical usage, and preservation of the native structure and functionality of sensitive proteins. However, it generally results in low protein yields [76], as it is ineffective for extracting poorly water-soluble proteins like globulins, which are predominant in legumes [4]. Additionally, further purification steps are often required to concentrate the extracted proteins. Langton et al. soaked faba beans in water overnight and modified the pH of water slurry to 8 with the use of NaOH (5 M), resulting in a product with a protein content of 43.4%, which was lower than the 61.7% protein in a product extracted with alkali [77]. Peyrano et al. obtained 26% protein from defatted cow pea flour [78], which was isolated via water extraction followed by alkaline extraction and then lastly followed by isoelectric precipitation [79].

#### 3.1.2. Alkaline Extraction

Alkaline extraction employs strong alkali (such as sodium or potassium hydroxide) to enhance the yield of protein extracted via increasing pH followed by precipitation at their isoelectric point. At alkali conditions (pH = 8–10), the protein interactions like van der Waals forces and hydrophobic interactions between protein molecules are destroyed by strong electrostatic repulsions and increase the protein solubility [39]. This method offers high extraction yields and is particularly suitable for globulins. Its simplicity, low cost, and scalability make it attractive for industrial applications. However, the use of high pH may lead to protein denaturation, reduced solubility, and loss of functional properties. In some cases, undesirable reactions such as the formation of lysinoalanine can occur. Additionally, this method requires extensive water use and generates alkaline wastewater, raising environmental concerns. After using different concentrations of NaOH to extract ganxet beans, Lafarga et al. observed that a 0.4 M NaOH solution (pH 12.94) resulted in an isolated material yield of 24.02% and a protein content of 50.17% [42]. Alkaline extraction combined with isoelectric precipitation is a common procedure, as the alkaline pH enhances protein solubilization, while the isoelectric point represents the pH at which proteins precipitate due to their minimal solubility. Alkaline extraction of defatted acacia seed protein was conducted at pH = 10, and after centrifugation the pH of supernatant was adjusted to pH = 4.0 to increase the recovery of protein [32]. Faba bean protein extracts obtained at pH = 9.5, followed by isoelectric precipitation, resulted in protein isolates with 77% protein and a solubility of 66.53% [51]. Ganxet beans yielded 51.56% protein after isoelectric precipitation, which was higher than the 24.02% obtained through alkaline extraction alone [42].

#### 3.1.3. Salt Extraction–Dialysis

Salt extraction–dialysis consists of two procedures: (a) solubilization of salt-soluble proteins with salt solutions at a neutral pH and moderately warm temperature (up to 35 °C), followed by (b) protein recovery with the use of membranes (dialysis) or precipitation at a low temperature (1–4 °C) [4]. This method is particularly effective for extracting globulins and better preserves protein structure and functionality compared to extreme pH methods. Its main advantages include the use of milder conditions, making it suitable for functional food applications. However, it is time-consuming and difficult to scale up due to the lengthy dialysis process. The overall protein yield may also be lower compared to alkaline extraction if conditions are not optimized, and water consumption can be significant. Stone et al. extracted protein from three varieties of pea via salt extraction–dialysis by mixing defatted pea flour with sodium phosphate buffer (pH 8.00) containing 6.4% KCl, followed by separation at 4 °C and dialysis with a 6–8 kDa cutoff membrane [19].

#### 3.1.4. Micellization Precipitation

The steps in micellization involve the mixing of proteins with salt solutions at neutral pH and room temperature, followed by centrifugation and precipitation at cold temperature (4 °C). The goal of micellization is to minimize protein denaturation and to preserve the functional properties of the proteins by maintaining their native structure and solubility [70]. However, this method is technically demanding, as it requires precise control of pH, ionic strength, and temperature. Additionally, it may not be suitable for proteins tightly bound to the cellular matrix, and its scalability for industrial applications remains limited. Despite its potential, micellization precipitation is less commonly used due to its complexity and relatively low overall efficiency. Compared to alkaline extraction, micellization is considered less aggressive in terms of lower protein denaturation and ordered globular structure [80]. To obtain protein isolates from hyacinth beans, the defatted flour was mixed with a sodium phosphate buffer containing 6.4% KCl [26]. Micellized mung bean protein isolate (pH = 6.0) was reported to contain the highest solubility compared to protein obtained via alkali extraction (pH = 8) [80].

**Table 2:** The sources, methods of extraction, yield, and the protein content in the extracted products by conventional methods.

Sources	Extraction Methods	Protein Yield (%)	Protein Content (%)	Reference
Hyacinth bean	Isoelectric precipitation	68.89	84.41	[26]
Hyacinth bean	Micellization precipitation	72.60	87.78	[26]
Hyacinth bean	Salt extraction–dialysis	66.17	71.09	[26]
Acacia coriaceae	Alkaline extraction followed by isoelectric precipitation	53.4	31.7	[32]
Acacia victoriae	Alkaline extraction followed by isoelectric precipitation	65.2	44.6	[32]
Faba bean	Alkaline extraction	16.41	89.88	[25]
Carob	Alkaline extraction	96	46	[81]
Madras thorn	Alkaline extraction	48.66	85.17	[50]
Defatted soy grit	Enzyme-assisted extraction coupled with alkaline extraction	45.93	46.16	[50]
Peanut	Alkaline extraction followed by ultrasound	50	90	[44]
Faba bean	Alkaline extraction	61.7	24.4	[77]
Faba bean	Soaked or water	43.4	21	[77]
Ganxet bean	Alkaline extraction followed by isoelectric precipitation	51.56	50.17	[42]
Lima bean	Water–alkaline extraction	10.67	88.01	[35]
Pea	Micellization precipitation	31.1	87.8	[19]
Pea	Salt extraction–dialysis	68.2	76.1	[19]
Chickpea	Enzyme-assisted extraction	21.42	92.89	[82]

### 3.2. Non-Conventional Extraction Methods

Recently, non-conventional methods for protein extraction have been increasingly developed and utilized due to the numerous advantages they offer compared to traditional extraction techniques. These emerging technologies offer several benefits, including higher extraction efficiency, reduced processing time, lower environmental impact, and the potential to preserve or even enhance the functional and nutritional properties of proteins. One of the key distinguishing features of these non-conventional methods is their ability not only to extract proteins but also to physically modify their structure. Unlike traditional methods, which may preserve the protein’s native conformation, non-conventional techniques can alter the protein’s secondary and tertiary structures [83]. These modifications often result in improved protein functionality, such as better solubility, emulsifying, foaming, or gel-forming abilities. Common physical modification techniques include high temperature, high pressure, ultrasonication, microwave treatment, irradiation, and pulsed electric fields. These methods induce changes in protein conformation and aggregation, which can enhance protein performance in various food and industrial applications [83]. By modulating protein structure, these methods can potentially improve both the nutritional profile and the functionality of the extracted proteins. As a result, they represent a promising alternative to conventional approaches, aligning with the growing demand for sustainable and innovative food processing solutions. Table 3 lists raw materials used for protein extraction through emerging technologies, along with the impact of these methods on protein yield, functionality, and structure in comparison to traditional extraction methods.

#### 3.2.1. Enzymatic Extraction

A common strategy to select protein from plants consists of the disruption of cell wall components, like pectin and cellulose. Carbohydrases (celullase, pectinase, hemicellulase) are enzymes with specificity to hydrolyze polysaccharides and subsequently increase the yield of extracted legume protein. Proteases (papain, Neutrase, Alcalase) hydrolyze protein-generating peptides. Peptide structures contain 2–50 amino acids, while protein structure has more complexity, reaching 50 or more amino acids. Carbohydrases are used to pretreat the legume matrix before the alkaline extraction or/and after isoelectric precipitation to increase the yield of proteins in legume matrices [13,29]. A commercial cocktail of carbohydrases coupled with alkali enhanced the extraction yield of defatted soy grit protein by 45.93%, which was significantly higher than 35% obtained by enzyme aqueous extraction, and next to the 41.03% yield obtained after alkaline extraction for 3 h [84]. Alkaline extraction of chickpea flour assisted with arabinofuranosidase and a mix of cellulase and xylanases recovered protein by approximately 21%, which was higher than the 15.76% recovered by single alkaline extraction. Also, the quality of proteins extracted by these enzymes was reported to be superior compared to alkaline extraction, in terms of solubility and water- and oil-holding capacity [67].

#### 3.2.2. Ultrasound-Assisted Extraction (UAE)

US is widely recognized as an eco-friendly and efficient alternative to conventional food processing methods in the food industry [85]. This technology is classified based on frequency and intensity [86]. High-frequency, low-intensity US (f > 100 kHz) is suitable when preserving the physical and chemical properties of materials is critical. In contrast, low-frequency, high-intensity US (20 kHz ≤ f ≤ 100 kHz) interacts more directly with the material, inducing physical, chemical, and mechanical changes [86]. US enhances the extraction of various compounds from plant matrices, including polyphenols, carotenoids, anthocyanins, essential oils, proteins, polysaccharides, pigments, and iridoids [86]. For protein extraction from plant-based sources, high-power ultrasound (frequencies of 20–100 kHz and power levels of 100–1000 W/cm^2^) have been reported as effective, increasing extraction yields by approximately 20–50% [45,87]. This efficiency arises from acoustic cavitation, which disrupts plant cell walls, enhancing solvent penetration and improving mass transfer of analytes into the solvent. The creation and collapse of cavitation bubbles produce extremely elevate local temperatures (above 5000 K) and pressures (approximately 500 atm), leading to the formation of free radicals through the sonolysis of water [88]. Because of that, an ice bath is usually used to control the temperature, avoiding the thermal degradation of thermosensitive compounds. In this sense, ultrasound-assisted extraction (UAE) offers several advantages: it is selective, versatile, and cost-effective, requiring less solvent and delivering high yields in a short time. Additionally, its operation at low temperatures minimizes protein thermal denaturation, thereby preserving or even improving functionality [46]. UAE can also be integrated with other extraction methods, such as alkaline extraction/acid precipitation, enzymatic processes, pressurized fluid extraction, pulsed electric field (PEF), or microwave-assisted extraction (MAE), to further enhance efficiency and yields. In terms of disadvantages, US-probe techniques face scale-up challenges, mainly due to inconsistent processing conditions [89]. US leads to structural alterations on the protein surface due to the cavitation phenomenon, which generates high local pressure and shear forces on the sample [83]. These forces induce conformational changes in protein structure, thereby modulating their functional properties. The intensity of ultrasonication plays a critical role in both the efficiency of extraction and the extent of protein modification. At higher US power levels (above 120 W), a significant reduction in protein particle size has been observed [90]. This size reduction can improve protein solubility and limit the formation of aggregates, which is beneficial for maintaining desirable functional properties such as emulsifying and foaming capacities. On the other hand, lower US intensities may disrupt hydrophobic interactions within protein molecules, increase molecular mobility, and promote protein aggregation [82]. In addition, the US alters protein’s physicochemical and functional properties, particularly the secondary structure, exposing hydrophobic amino acid residues and leading to changes in protein functionality [87]. These changes, depending on US parameters and raw material composition, can enhance or diminish functional properties such as emulsification, gelling, and solubility, especially under high power and prolonged sonication [87]. Additionally, US can modify amino acids by reacting with sulfhydryl and phenolic residues, forming new covalent bonds between proteins. Both US probes and US baths have shown effective results in protein extraction and in enhancing protein functionality [89]. Key factors influencing protein extraction yield in UAE include treatment time, pH, temperature, and US intensity. Table 3 presents legume sources where US has been applied for protein extraction. It is worth noting that increased protein yield does not always correlate with improved functionality, highlighting the importance of balancing yield and functionality. Protein functionality changes are primarily related to alterations or denaturation in the secondary structure. The secondary structure, comprising α-helices, β-sheets, β-turns, and random coils, depends on amino acid sequences and backbone interactions. Ultrasonication can disrupt protein interactions, altering these structures. These structural modifications may expose additional enzymatic hydrolysis sites, enhancing the degree of hydrolysis and, consequently, the biological activity of the protein. Zhu et al. (2018) [91] reported that ultrasound treatment led to a 22% increase in water solubility, a 26% increase in the emulsifying activity index, and a 41% increase in the emulsifying stability index of walnut protein. These improvements were attributed to the ability of high-intensity US to disrupt the physical bonds between and within spherical protein molecules, resulting in partial unfolding and dissociation of the protein structure. Similarly, Hu et al. (2013) [92] observed a positive effect of US on the water-holding capacity and gelling properties of soy protein isolate, which was also linked to US-induced structural modifications. Most studies in Table 3 did not observe differences in SDS-PAGE profiles, indicating no changes in primary structures (linear amino acid sequences). However, tertiary and quaternary structures of proteins can be partially or completely unfolded under high-intensity ultrasound, as the technique disrupts hydrogen bonds and hydrophobic interactions, thereby altering overall protein conformation [83]. This agrees with the changes in fluorescence intensity post-sonication observed by [89,93]. The effects of US vary depending on the raw material composition, highlighting the need for studies across different materials to establish optimal extraction conditions. For instance, Table 3 shows that US positively affected protein extraction yields in black beans [41], lentils [41], and cowpeas [45], while Tang et al. [89] found no significant differences between conventional and US methods (probe system and bath) for pea proteins. In contrast, Byanju et al. [43] observed a decrease in extraction yield for chickpeas proteins after sonication (20 kHz, 315 and 390 W/cm^2^). This reduction was attributed to the high fat content (7%), which forms protein–lipid interactions that inhibit protein dissolution, and to the high carbohydrate content (~66%), which contains cellulose and non-cellulosic polymers that lower free water availability and increase viscosity, hindering protein extraction. These findings suggest that fractionating the raw material, starting with the extraction of non-polar compounds, followed by carbohydrates, could improve protein recovery. Additionally, variations in results, even within the same raw material, emphasize the impact of extraction conditions, equipment setups, and the properties of the material regarding its harvest. For faba beans, Badjona et al. [25] reported increased protein yield using UAE at 123 W and 20–25 °C (pH 11, 41 min, S/F = 15:1, solvent/feed ratio) compared to conventional methods. In contrast, Suchintita et al. [38] found no significant difference between UAE (20 kHz, 100% amplitude, 57.58 W/cm^2^, 20 min) and conventional extraction (500 rpm, room temperature, 18 h). Interestingly, they also observed that water-based extraction was more effective than alkaline extraction (pH 10) for the extraction of faba bean proteins. These findings underscore the complexity of UAE and the interaction between raw material composition and processing parameters. Future studies could focus on optimizing UAE conditions and exploring strategies tailored to different plant matrices to enhance both yield and functionality.

#### 3.2.3. Microwave-Assisted Extraction (MAE)

Microwave (MW) energy, a form of non-ionizing electromagnetic radiation with frequencies ranging from 0.03 GHz to 300 GHz, penetrates food materials and generates heat through mechanisms such as ionic conduction and dipole rotation [6]. This energy disrupts hydrogen bonds, promotes ion movement, and increases the porosity of biological structures, enhancing solvent penetration and facilitating the extraction of target compounds. Since plant matrices typically contain water (a polar solvent), MW energy is rapidly absorbed, leading to internal superheating, rapid evaporation, high intracellular pressure, and cellular disruption [94]. These combined effects promote the efficient release of intracellular components, reducing both extraction time and solvent consumption compared to conventional methods [46]. Additionally, MW systems are compact, cost-effective, and energy-efficient, making them particularly appealing for industrial applications [95]. The MAE process is influenced by various factors, including cell wall permeability, solvent selection, S/F, applied pressure, extraction duration, temperature, and the configuration of the extraction system, whether closed or open [6]. This technique has been widely applied to extract compounds such as phenolics [96], pigments [97], and polysaccharides [98]. However, its application in protein extraction remains limited. Kargar and Sourki (2024) [99], reported that pretreatment with MW irradiation (900 W, 5 min) improved the protein extraction efficiency by up to 175.3% and protein content by up to 1779.12 mg/kg of kabuli chickpea aquafaba. As observed with the UAE method, studies have shown that MAE not only enhances protein recovery but also improves the nutritional and functional properties of proteins, including solubility, emulsifying capacity, foaming ability, and digestibility [22,44,100], thereby increasing its potential for applications in the food industry. These improvements are mainly attributed to MW-induced changes in the secondary and tertiary structures of proteins. However, high MW power (1000 W, 50%) may cause significant protein denaturation, altering the protein structure from highly ordered configurations, such as α-helix and β-sheet, to disordered structures, including random coil and β-turn [100]. The main mechanism of MW fields on changes in food protein structure is assumed to be caused by carbon-centered free radicals. The polar groups of protein molecules can absorb energy and the kinetic energy can generate free radicals under MW radiation. These free radicals are active and can interact with amino acid residues, accompanied by the orderly arrangement of ions under MW heating, leading to changes in the secondary and tertiary structures [101]. MW has a distinct influence on the secondary and tertiary structures of protein under different treatment conditions. Recent studies have shown that non-covalent bonds of protein molecules can be broken down by MW heating, such as disulfide bonds and hydrogen bonds, which may facilitate the unfolding of protein structures, resulting in the subsequent changes in protein structures. Qin et al. (2016) [102] reported that MW pretreatment could destroy the secondary structure of soybean protein isolate and wheat gluten mixtures by facilitating the formation of disulfide bonds, as their results showed that at a maximum MW power of 700 W, the contents of α-helix and β-turn were increased by 1.83% and 1.71%, respectively, while β-sheet content was decreased by 3.54%. Zhang et al. (2025) [103] reported that MW treatment (up to 60 s) promoted the formation of ordered lamellar structures in soybean 7S protein through thermal and non-thermal effects, inducing aggregation via non-covalent interactions and disulfide bonds. Prolonged exposure, however, led to structural disruption and fragmentation [103]. Additionally, a reduction in fluorescence intensity was observed at 60 s, likely due to aggregation-induced encapsulation of aromatic residues (e.g., tyrosine, tryptophan), which stabilized the tertiary structure and reduced fluorescence. Additionally, Li et al. [104] reported a 24.7% reduction in the allergenicity of microwaved soy protein isolate used in infant formula, which was linked to alterations in its secondary structure. The influence of MAE parameters on protein extraction appears to be highly dependent on the raw material used. For example, Tang et al. [100] observed no significant difference in lupin protein yield when increasing MW power from 500 W to 1000 W during a 10 min extraction. However, SDS-PAGE analysis revealed the increased intensity of protein bands (~98 kDa) and FTIR showed changes in the secondary structure, indicating structural alterations induced by the MW process. A negative effect of increased MW power (from 400 to 800 W) on jack bean protein yield was observed by Ajayi et al. [22]. To mitigate the denaturation effects on proteins, the literature recommends using low to moderate power levels (600–1000 W) and limiting extraction times to 60–120 s [105]. The MAE technique has also been studied in combination with the UAE method. For instance, Tang et al. [100], investigated the sequential application of UAE (100 and 200 W for 10 min), followed by MAE (500 and 1000 W for 10 min), for protein extraction from lupin. Their findings revealed that the combined approach yielded higher protein recovery compared to using each technique individually. This synergistic effect was attributed to the mechanical oscillation and stirring caused by ultrasonic cavitation, which led to more uniform MW heating and improved extraction efficiency. Furthermore, the study demonstrated that for all emerging technologies evaluated (UAE, MAE, and UAE + MAE) the protein content in the final isolate was lower compared to conventional alkaline extraction. The authors attributed this reduction to the simultaneous extraction of other bioactive compounds, such as polysaccharides and phenolics, which diluted the protein concentration in the final product. These findings highlight the importance of balancing protein yield and concentration to avoid increased costs associated with further purification steps. In this sense, optimization of process parameters is essential for achieving efficient and cost-effective protein extraction. However, contrasting results were reported by Ochoa-Rivas et al. [44], who investigated the extraction of peanut proteins using a combination of US (100% amplitude, 15 min) and MW (725 W, 8 min). No synergistic effect was observed in this case, reinforcing the need to optimize process parameters for different materials to establish potential relationships between the composition of the raw material, the effects of these technologies, and the impact of process parameters on the yield and quality of plant-extracted proteins.

#### 3.2.4. Pulsed Electric Fields (PEF)

PEF is another emerging and sustainable technology widely explored in food processing. This non-thermal technique has gained significant attention in recent years due to its low energy consumption, short processing time, and its ability to preserve thermosensitive components such as proteins by avoiding their thermal denaturation [106]. PEF operates by applying high-intensity electric pulses (in the range of kV·cm^−1^) between two electrodes within a treatment chamber [107] in short-duration pulses (ranging from nanoseconds to milliseconds). This voltage generates an electric field whose intensity is determined by the voltage applied and the distance between the electrodes. When the electric field reaches sufficient intensity, it induces electroporation, resulting in the formation of micropores in cell membranes and enhancing their permeability [107]. This enhanced permeability enables a higher mass transfer of intracellular components, enabling the selective extraction of these compounds. The two most frequently produced pulse waveforms are square pulses and exponentially decaying pulses, which can be generated in monopolar or bipolar configurations. Voltage, current, frequency, and waveform signals are observed and captured using digital data acquisition tools, such as an inline oscilloscope [108]. The efficiency of PEF extraction depends on several factors, including the electric field intensity, specific energy, treatment duration, temperature, and properties of the material being treated, such as pH, conductivity, and matrix characteristics [109]. While PEF can induce structural and functional modifications in food proteins, low-intensity treatments typically produce limited effects compared to thermal processing, as the temperatures reached are not sufficient to trigger protein denaturation [101,110,111]. However, excessively high electric field intensities can disrupt intra- and intermolecular electrostatic interactions in peptides, potentially causing macroscopic changes that may impact the quality of foods [106]. In addition to enhancing extraction rates and yields, PEF has been shown to preserve and modify the biological properties of extracted proteins, such as antioxidant activity and allergenic potential [106,107,112]. It can also inactivate undesirable microorganisms and enzymes [107]. During PEF processing, the application of high-intensity electric fields induces polarization of molecules by interacting with the dipole moments of peptides. This interaction can ionize carboxylic (-COOH) and amino (-NH_3_^+^) groups, altering the surface charge distribution of proteins [108]. As a result, repulsive and attractive electrostatic forces are modified, promoting unfolding of the tertiary structure, exposure of hydrophobic residues, and subsequent protein–protein interactions. These effects can lead to aggregation and disruption of ordered secondary structures, such as the α-helix, ultimately influencing the protein’s solubility and functional behavior [108]. In summary, PEF not only enhances the extraction of proteins and other bioactive compounds but also induces structural and functional modifications depending on the applied intensity, making it a promising approach for developing high-quality functional foods. The main drawback of PEF technology lies in the high cost of the equipment, which requires a substantial initial investment. Nonetheless, its low operational costs can offset this initial expenditure over time. Another limitation of PEF is its dependency on the conductivity of the medium being treated. Elevated conductivity levels can diminish both the efficiency and precision of the electroporation process [113]. Lai et al. [114] observed that increasing the electric field intensity from 0.8 to 0.9 resulted in a significant improvement in protein extraction yield. This enhancement was attributed to the higher energy input, which more effectively facilitates the disruption of cell membranes compared to lower electric field intensities, and this can be due to the higher energy input, which facilitates the rupture of the cell membrane more easily than lower electric field intensities. The pulse number, referring to the total number of pulses applied during PEF treatment, is another critical parameter, as PEF processes typically involve a series of pulses [112]. Some studies have demonstrated that the extraction yield increases as the pulse number increases [115,116]. Additionally, longer pulse repetition times have been associated with more efficient membrane poration [112]. Although PEF technology shows great potential for assisting in protein extraction and studies highlight legumes as a significant source of protein, the number of investigations into PEF for legume protein extraction remain limited, as shown in Table 3. This can be attributed to the fact that PEF is still an emerging technology, with access to the necessary equipment restricted to a few research groups. Additionally, operational challenges, such as optimizing system parameters, contribute to this limitation. For instance, the distance between the electrodes is crucial, particularly for the extraction of large molecules like proteins, as it influences the electric field intensity. Furthermore, the pH of the extraction medium plays a significant role. Protein extractions are typically conducted under alkaline conditions (pH > 8) to enhance solubility by increasing the net negative charge of protein molecules and promoting electrostatic repulsion. However, higher pH levels also increase the ionic strength and electrical conductivity of the medium. In the context of PEF, a higher conductivity reduces the dielectric breakdown threshold and leads to excessive current flow, which can trigger safety mechanisms or result in equipment shutdown due to overheating or energy overload. Moreover, legumes with naturally high mineral content such as sodium, potassium, or calcium can further increase the conductivity of the extraction medium, aggravating this issue. In our research group, we have observed this negative effect of the raw material composition submitted to PEF treatment in whole lupin seed (*Lupinus angustifolius* L.) and in other biological matrices like seaweed (*Palmaria palmata*). Similar challenges could arise with mineral-rich legumes such as faba beans, cowpea or lentils, which are rich in iron, zinc, potassium, phosphorus, calcium, and sodium [110,111]. These challenges emphasize the need for further research to optimize PEF parameters for legume protein extraction and expand its applicability in this field. One of the few studies investigating the effects of PEF on legume proteins was conducted by Li (2012) [117] who reported that PEF treatment increased the proportion of β-sheet structures in soybean protein isolates, which was attributed to protein aggregation and the formation of hydrogen bonds between β-sheet lamellae. Additionally, significant changes in sulfhydryl groups and hydrophobicity were observed, as indicated by alterations in the vibrational peaks of disulfide bonds and tyrosine frequencies. Another relevant study by Lai et al. [114] evaluated the effects of PEF and a combined PEF + UAE approach on chickpea protein extraction. The combination of PEF (30–150 s, 0.7–1.1 kV/cm) and UAE (5–25 min, 240–400 W) significantly enhanced both protein extraction yield and functional properties, such as solubility, emulsification, and foaming of chickpea protein isolate compared to conventional alkaline extraction. Notably, the PEF-US combination increased protein yield by 47.28% compared to the conventional method and by 22% and 20% compared to PEF and UAE individually, respectively. This improvement was attributed to the synergistic effects of the two technologies: PEF treatment effectively disrupts cell walls, facilitating protein release, while the cavitation effect of US generates shock waves through the formation and collapse of microbubbles, enhancing the interaction between proteins and the surrounding solutes.

#### 3.2.5. Ohmic Heating (OH)

Ohmic heating (OH), also referred to as Joule heating, electrical resistance heating, or direct electrical resistance heating, is a cutting-edge technology in the food processing industry [118]. OH is a process of heating food by passing an electric current. It relies on the application of moderate electric fields (MEFs), typically below 1000 V·cm^−1^, resulting in heat dissipation [119]. The energy is dissipated directly into the food, and electrical conductivity is a key parameter in the design of an effective ohmic heater. OH generates heat within the food material through electrical resistance, leading to rapid and uniform heating [120]. Thermal and non-thermal effects are the primary mechanisms driving OH extraction. Thermal effects enhance tissue softening, increase solute solubility, and improve mass transfer coefficients. On the other hand, nonthermal effects involve electropermeabilization and electroporation, which facilitate the diffusion of solutes from the food matrix into the solvent [121]. OH enables the extraction of various compounds with significantly reduced environmental impacts. It requires less water and solvents, generates minimal waste, and results in higher extraction yields, superior extract quality, and shorter processing times [120]. OH helps maintain proteins’ nutritional and functional properties by minimizing thermal degradation [122,123]. The impact of ohmic heating on protein denaturation and aggregation, which affects both non-covalent and covalent bonds, has significant implications for the techno-functional properties of proteins. This underscores the necessity of adjusting processing conditions to fully leverage the benefits of ohmic heating [123]. OH can improve the water- and oil-holding capacities of proteins, which are crucial for their emulsification and foaming properties. It also increases the solubility of legume proteins. Studies show that the solubility of pea protein increased by approximately 240% after being treated using OH at 150 °C [124]. Despite its many benefits, ohmic heating faces challenges like viscosity, electrical conductivity, and fouling deposits. To optimize extraction and mitigate these issues, it is crucial to carefully control factors such as electrical field intensity, treatment temperatures, and heating strategies [125]. Studies on the use of OH for the extraction of legumes are very limited, and most of them are focused on the modification of proteins extracted using other technologies. Some applications of OH for the extraction of protein from legumes are summarized in Table 3.

#### 3.2.6. Subcritical Water Extraction (SWE)

Subcritical water extraction (SWE) uses only water at a temperature between 100 and 374 °C and at sufficiently high pressure to maintain water in a liquid state [126]. Maintaining water in this condition dramatically changes its physical and chemical properties, such as low viscosity, low surface tension, high diffusion, low dielectric constant, and increased ionic products (Kw) [127]. Subcritical water also acts as both a solvent and catalyst, aiding in the extraction and hydrolysis processes essential for maximizing protein yields [128]. Thus, the catalytic medium created in SWE can be used to hydrolyze complex biopolymers, such as proteins, to produce bioactive peptides and amino acids [129]. This process has several advantages over conventional extraction methods, such as lower solvent consumption, high extraction yield, fast processing time, and no use of organic solvents [130].

Specifically, within SWE, higher temperatures facilitate the disruption of intracellular structures, resulting in enhanced protein solubility and recovery. This interplay of heat and pressure enhances the release of proteins while potentially preserving their functional properties, which is a critical consideration in food applications. The processing temperature is the most important parameter. Increasing the temperature results in a higher hydrolysis yield of protein and increases the generation of lower molecular weight fractions. SWE enhances the emulsifying properties of legume proteins, making them more effective in stabilizing oil-in-water emulsions. It can also lead to partial hydrolysis of proteins, which can improve digestibility and functionality [131]. Nevertheless, the processing conditions can be optimized to generate hydrolysates of desired functionalities [132]. Some applications of SWE for the extraction of protein from legumes are summarized in Table 3.

#### 3.2.7. Deep Eutectic Solvents (DES)

Deep eutectic solvents (DESs) are mixtures of a hydrogen bond donor (HBD) and a hydrogen bond acceptor (HBA). They have low melting points, often lower than their individual components, making them ideal for extracting heat-sensitive compounds like proteins [133]. DESs allow for efficient extraction while preserving the proteins’ bioactivity and structure. DESs disrupt non-covalent interactions, such as hydrogen bonds and van der Waals forces, within legume matrices, allowing for selective protein solubilization without breaking peptide bonds or altering secondary or tertiary structures. Studies have shown DESs are effective in extracting legume proteins. For example, Chen et al. [134] optimized soy protein extraction using DESs, and highlighted that DESs can improve yield and functional properties compared to conventional methods. Proteins extracted with DESs retain beneficial properties like emulsifying and foaming abilities, which are valuable in food applications. However, some combinations of DESs potentially have toxicity and, to address this problem, DESs have recently been further developed into natural deep eutectic solvents [135]. NADES composed exclusively of natural compounds have garnered attention for their additional advantages in extraction protocols. This category covers the deep eutectic solvents that are made of primary metabolites such as organic acids, amino acids, sugars, polyols, and choline derivatives [136]. These solvents not only provide high solubility for polar compounds but also feature biodegradability and biocompatibility, which are important considerations in food science applications [137]. DESs are essential for protein extraction, creating a DES–protein mixture through aggregation and surrounding phenomena. This method enables efficient protein extraction while preserving their structural integrity, making it ideal for nondestructive extraction of bioactive compounds. However, challenges such as low extraction efficiency due to high viscosity and limited selectivity for specific proteins persist [138]. In a recent study, Hewage et al. [139] used choline chloride and a glycerol-based DES to extract protein from faba bean, and they obtained a higher protein yield and recovery rate than alkaline extraction. In another study, Chakravorty and Das [140] used several choline chloride-sugar-based NADES to extract protein from tree beans, and protein extracted using sorbitol-based NADES showed superior emulsification (50.42%) and stability (42.55%) compared to other formulations.

#### 3.2.8. Dry Fractionation

The dry fractionation process operates primarily through the air classification of milled legume flour, whereby lighter protein-rich particles are separated from denser starches [141,142]. Dry fractionation utilizes physical processes such as milling and air classification to separate protein-rich fractions from legumes without using additional solvents or chemicals, thus offering a more sustainable alternative to traditional wet fractionation methods [143,144]. Moreover, this process preserves the native functionality of proteins, which is otherwise lost in wet fractionation methods [141]. Research has indicated that proteins extracted through dry methods maintain favorable characteristics such as emulsifying capabilities, water-holding capacity, gelation, and solubility, which are vital for food applications [145,146]. Many legumes are characterized by high protein content, primarily composed of globulins and albumins, which contribute to their nutritional value. Dry fractionation has shown efficacy in preserving these essential proteins while also enhancing their functional properties [146,147]. Despite operational efficiencies achieved for the dry fractionation process, the oil content of the legumes remains the main factor that influences separation efficiency. The presence of high oil content can hinder effective protein separation. Legumes containing high amounts of oils, such as lupin and soybean, have been reported to be unsuitable for dry fractionation [148,149]. Thus, deoiling must be applied for legumes with high oil content. Deoiling improves the processability of the flours as well as their composition and particle size [148]. Hopf et al. [150] compared the techno-functional properties of wet and dry fractionated protein from several legumes and found that dry fractionated proteins have high solubility, higher emulsification capacity, and a lighter color than wet fractionated proteins, demonstrating significant potential application in food. In another study, Schlangen et al. [145] used dry fractionation to produce functional fractions from mung bean, yellow pea, and cowpeas. The protein contents of the fractions ranged from 42 to 58%, which were influenced by the classifier wheel speed and material source. They also reported that water-holding capacity positively correlated with mung bean and cowpea protein content but negatively correlated with yellow pea. This showed that differences in functionality were related to protein characteristics rather than just protein content [145]. Examples of dry fractionated legumes and their techno-functional properties are shown in Table 3.

**Table 3:** Protein extraction using emerging technologies: legume sources, protein content, and impact on functionality.

Sources	Extraction Methods	Extraction Conditions	Protein Yield (%)	Protein Content (%)	Effect on Functional Properties	Comments	Ref.
Black beans	UAE (US bath) 37 kHz, 320 W	Solvent: Tris–HCl buffer pH = 9 t = 20 min S/F = 5:1 T = 25 °C	9.7	-	WHC: +297% EAI: +13% Tgel: −3.5 °C	IP: 3.5 No changes in primary structure	[41]
Lentils	UAE (US bath) 37 kHz, 320 W	Solvent: Tris–HCl buffer pH = 9 t = 20 min S/F = 10:1 T = 25 °C	7.6	-	WHC: +18% FC: +30% EAI: +10% ESI: +163% Tgel: −3.7 °C	IP: 5.0 No changes in primary structure	[41]
Faba beans	UAE (probe system) 123 W	Solvent: water pH = 11 t = 41 min S/F = 15/1 T = 20–25 °C	19.7 (+20%)	93 (+3%)	WHC: +18% OHC: +20% FC: −7%	IEP = pH 4.0 (1 N HCI) No changes in primary structure; secondary structure modified; and isolates were thermally stable	[25]
Faba beans	UAE (probe system) 20 kHz, 100% amplitude, 57.58 W/cm^2^ power intensity	Solvent: water t = 20 min S/F = 10/1 T = 20–25 °C	10.9 (ns)	84 (ns)	-	IEP = pH 4.5 (1 N HCI) SEM showed low adherence and few protein bodies due to greater protein leaching	[38]
Faba bean	UAE (probe system) 20 kHz, 100% amplitude, 57.58 W/cm^2^ power intensity	Solvent: water pH = 10 (0.1 M NaOH) t = 20 min S/F = 10/1 T = 20–25 °C	12.6 (ns)	80 (ns)	-	IEP = pH 4.5 (1 N HCI)	[38]
Cowpea pulse crop	UAE (probe system) 100 and 200 W	Solvent: water pH = 9 (1 N NaOH) t = 5–20 min S/F = 10/1 T = 25 °C	59 (+85%), 200 W and 10 min	-	Solubility: +20% (200 w/10 min) FC: +18% (200 w/10 min) FS: +100% (200 w/10 min) WHC: +20% (200 w/10 min) OHC: +58% (200 w/15 min) EAI: +35% (200 w/10 min) ESI: +55% (200 w/10 min)	IEP = pH 4.5 (1 N HCI) Sonicated samples presented higher zeta potential and smaller particles	[45]
Raw pea powder	UAE (probe system) 750 W, 30% amplitude	Solvent: water pH = 9 (1 M NaOH) T = 10 min S/F = 9/1 T = 25 °C	83% (+15%)	87.5 (+7%)	FC: +19% FS: +23% WHC: +38% OHC: +7% EAI: +11% ESI: +7% LGC: −18%	IEP = pH 4.5 Intact primary structure; secondary structure changed; smaller pea protein particles	[37]
Peanut Flour	UAE (probe system) 24 kHz, 20 and 100% amplitude	Solvent: water pH = 9 (50% NaOH) t = 15–40 min S/F = 10/1	50–67% (+119%, 100% amplitude and 40 min)	~90 (ns)	WAI: +700% WSI: −66% FC: +34% FS: −21% EA: −5% In vitro digestibility: +3%	IEP = pH 4.5 (15% HCl)	
Pea protein	UAE (US bath), 25 kHz UAE (probe system) 500 and 1000 W	Solvent: water pH = 10 (1 M NaOH) T = 60 min for US bath T = 30 min for US probe S/F = 20/1	12% for UAE bath (ns) 14% for US probe (ns)	79% for UAE bath (ns) 73–75% for US probe (ns)	-	IEP = pH 4.5 No significant primary structure changes; US probe altered secondary structure; tertiary structure changes noted via reduced fluorescence	[89]
Lupin	UAE (probe system) 50 and 100 W	Solvent: water pH = 10 (1 M NaOH) t = 10 min S/F = 20/1	17.43% for 100 W (+25%) 16.41 for 50 W (+18%)	82.5% for 100 W (−15%) 85.97% for 50 W (−11%)	No improvements in solubility	IEP = pH 4.5 (1 M HCl) Protein band intensity changed at ~98 kDa; particle size increased	[100]
Chickpea	UAE (probe system 325 W	Solvent: water pH = 9 (1 M NaOH) t = 10 min S/F = 10/1	11% (+20%)		Solubility: +8% EA: +96% FA: +18%	IEP = pH 4.0 (1 M HCl) Surface morphology looser and fragmented; tertiary structure altered; increased hydrophobicity	[114]
Jack beans	MAE, 400 W, 600 W, and 800 W	Solvent: water pH 9.0, 10.0, and 11.0 (1.0 M NaOH) t = 5 min S/F = 10/1	45.7–70% (+up to +53%) Maximum yield at 400 W and pH 10	68–80% (up to +17%)	Solubility: −43% at 400 W and pH 10, and increased +3.9% at 400 W and pH 11 EAI = up to +108% (400 W pH 9) ESI = up to +300% (800 W, pH 9) FC = +33% (400 W, pH 9) FS = +8% (400 W, pH 10) WHC = 308% (400 W, pH 9) OHC = +30.6% (400 W, pH 9)	IEP = pH 4.6 (1.0 M HCl) Particle size increased in microwaved samples; FI increased at 400–600 W, then decreased at 800 W	[22]
Peanut Flour	MAE, 725 W and 8 min	Solvent: water pH 9.0 (50% M NaOH) t = 2–10 min S/F = 25/1 and 10/1	55% (+77%),	93%	WAI: +900% WSI: −68% FC: +15% FS: −26% EAI: +5% In vitro digestibility: +2%	IEP = pH 4.5 (15% HCl)	[44]
Lupin	MAE, 1000 W	Solvent: water pH = 10 (1 M NaOH) t = 10 min S/F = 20/1	18.2% (+30%)	83.57% (−14%)	No improvements in solubility	IEP = pH 4.5 (1 M HCl) Protein bands intensified, likely due to submicron aggregation; particle size increased	[100]
Lupin	MAE (500 and 1000 W) + UAE (probe system, 100 and 200 W)	Solvent: water pH = 10 (1 M NaOH) t = 10 min S/F = 20/1	22.3% (+60%, 200 W UAE + 1000 W MAE)	76.4% (−21%) (−21%, 200 W US + 1000 W MW)	Solubility was significantly increased by almost +7% (UAE at 100 W and MW at 500 W MW)	IEP = pH 4.5 (1 M HCl) Particle size increased	[100]
Chickpea	PEF (87 s and 0.9 kV/cm)	Solvent: water pH = 9, (1 M NaOH) S/F = 10/1	11% (+20%)	-	Solubility = +9% EA = +61%	IEP = pH 4.0 (1 M HCl) Changes in tertiary structure; hydrophobicity increased	[114]
Chickpea	PEF (87 s and 0.9 kV/cm) + US (15 min and 325 W)	Solvent: water pH = 9 (1 M NaOH) S/F = 10/1	13.52% (+47%)	-	Solubility = +11% EA = +110% FA = 12%	IEP = pH 4.0 (1 M HCl) Changes in tertiary structure; hydrophobicity increased	[114]
Lentils	OH, 5 V/cm and 75 V/cm and 80 °C, 20 min	Solvent: water pH = 3 (1 MHCl) S/F = 1/50	-	-	-	Increased surface hydrophobicity Change in structure depends on the pH and electric field strength	[122]
Pea protein	OH, 13 V/cm and 50 V/cm	Solvent: water pH = 7 S/F = 1/50					
Soybean meal raw and deoiled	OH, 210 °C, 30 min, (raw); 200 °C (deoiled)	Solvent: water S/F = 1:5	44.4% (raw), 33.3% (deoiled)	-	-	-	[151]
Soybean flakes	SWE, 66–23 4 °C, 13–47 min	Solvent: water S/F = 1:3.3–1:11.7	Up to 59.2% at 66 °C, 20–40% at >100 °C	-	-	-	[152]
Soybean	Enzyme assisted SWE, 120 °C, 20 min	Solvent: water S/F = 1/10	59.3%	>80%	Increased solubility Change in hydrophobicity of the protein Increased emulsifying activity High interfacial activity	-	[153]
Defatted soy meal	SWE, 100–250 °C, 5 min	Solvent: water S/F = 1/40	52%	-	-	The emulsification and foaming capacity of the extract was highly dependent on the extraction temperature	[154]
Faba bean	DES	Solvent: (Choline chloride and glycerol) S/F = 1/10–1/30, 50–90 °C, 1–3 min	92.33%	65.42%	Increased secondary structure component, α-helix. A high β-sheet (38.61) was observed	-	[139]
Tree bean	NADES	Solvent: (Choline chloride-sorbitol) S/F = 1/20, 80 °C, 20 min	-	-	EA = 50.42% ES = 42.55%	-	[140]
Lentils	Dry fractionation	Air flow was set to 52 m^3^/h, feed rate 1.5–3.5 kg/h, internal pressure of the classifier 30–39 mbar	-	23.05–54%	- Particle sizes between 6 and 78 μm - Solubility = ~60% for all fractions	- The classified fractions showed differences in bulk rheology as well as changes in the kinetics of interfacial rheology. - Non-protein content also contributed to the functional properties	[155]
Yellow pea	Dry fractionation	Classifier wheel speed 4000 rpm, airflow 52 m^3^/h, feed rate 0.75 kg/h	63%	67%	-	The gel firmness of the protein-rich fractions was affected by their starch content.	[156]
Pea	Dry fractionation	Classifier wheel speed 3166 rpm, air flow 1325 m^3^/h, feed rate 300 kg/h	35–43.5%	85–87%	-	-	[157]

MAE: Microwave-assisted extraction; UAE: Ultrasound-assisted extraction; PEF: Pulsed Electric Field; OH Ohmic heating: SWE: Subcritical water extraction; DES: Deep eutectic solvents; NADES: Natural deep eutectic solvents. (): Values in parentheses in the protein yield and protein content columns indicate changes compared to conventional extraction methods: an increase (+, positive effect), a decrease (−, negative effect), or no significant difference (ns). S/F: Solvent to feed ratio. IEP: Isoelectric precipitation. WHC: Water-holding capacity; OAC: Oil-holding capacity; EAI: Emulsion activity index; ESI: Emulsion stability index; EA: Emulsion activity; FC: Foaming capacity; FS: Foaming stability.

## 4. Applications of Legume Protein for Food Development

Global population growth, the increasing demand for protein, and consumers’ preference for healthy, sustainable, and non-animal proteins produced sustainably have diversified the application of legume proteins in food product development. Legume proteins have functional uses in food applications, such as solubilization, emulsification, foaming, gelation, and dough formation, and are used in products like meat analogs, beverages, bakery, and pasta products [158]. The emulsifying and gelling properties of legume proteins are important for the development of plant-based dairy alternatives [29,159]. On the other hand, legume proteins’ water and oil absorption properties are essential in developing baked goods and meat analogs [29,160]. Other applications of legume proteins include the encapsulation [85] of bioactive compounds and the 3D printing of food products [158]. For the development of meat analogs, different combinations of legume proteins (pea, soy, and chickpea proteins) have been used with wheat gluten to create a fibrous structure and a balanced amino acid profile [161]. Legume proteins have also been used to prepare plant-based fat, which can mimic the appearance of beef adipose tissue but have a much softer texture than their real meat analogs [162]. Legume proteins are gaining recognition for their high nutritional value, providing a rich source of essential amino acids. Although many legumes are considered incomplete proteins due to their lower levels of sulfur-containing amino acids like methionine and cysteine, they can be effectively complemented with other protein sources, particularly cereals. This combination results in a well-balanced amino acid profile, which is vital for optimal nutrition [163]. Not only do proteins from legumes have health benefits, but also peptides derived from them have shown several biological activities, including antioxidant [164], anti-inflammatory, antihypertensive [165], and antidiabetic activities. These bioactive and functional properties of legume proteins have significantly contributed to modern culinary applications and molecular gastronomy. Molecular gastronomy is a scientific discipline that explores the physical and chemical transformations of ingredients during cooking. It aims to understand and innovate cooking methods to enhance food experiences, focusing on the sensory and cerebral interpretation of foods [166]. Legume proteins can be integrated into molecular gastronomy through various innovative techniques. Their excellent foaming and emulsification properties can be utilized in molecular gastronomy to create foams and emulsions. Their gel-forming property can be used to form hybrid hydrogels with improved texture and water retention, which is suitable for a fat replacer and bioactive ingredient delivery [159,167]. They can also be used in the spherification process to create spheres with unique textures and flavor, enhancing culinary experiences [167]. Although legume proteins have several applications in the development of food products, the presence of antinutritional factors in legumes adversely affects the digestibility and bioavailability of proteins. Therefore, they should be processed to minimize the antinutritional factors and increase their protein quality [168].

## 5. Technological Opportunities and Challenges in Legume Protein Extraction

### 5.1. Technical, Scalability, and Feasibility Aspects of Emerging Protein Extraction Technologies

One of the main challenges in applying emerging technologies for legume protein extraction lies in scaling up the process [169]. Laboratory-scale systems allow precise control over parameters; however, adapting them for continuous or industrial-scale production requires addressing issues such as treatment uniformity, energy efficiency, and process reproducibility. Small variations in operational conditions such as power intensity, temperature, extraction time, or solvent-to-solid ratio can significantly affect both protein yield and functionality at larger scales [170]. From an economic perspective, conventional extraction methods (e.g., alkaline, acidic, or solvent-based extraction) tend to be more affordable initially due to simple equipment and widely available reagents. However, these methods often incur hidden costs associated with solvent recovery, waste disposal, intensive energy, and prolonged processing times [171]. Over time, these factors can undermine their cost-effectiveness and sustainability. In contrast, emerging technologies often require higher initial investments, due to the need for specialized equipment. Nevertheless, they typically offer shorter processing times, reduced energy consumption, lower solvent usage, and improved protein quality, which may compensate for their initial costs. Techniques like OH or MAE for example, deliver energy directly to the sample, minimizing thermal degradation and reducing both extraction time and temperature requirements. Furthermore, green extraction approaches using environmentally friendly solvents or milder processing conditions reduce the use of toxic chemicals, lower waste management costs, and may facilitate regulatory compliance, especially in the food and pharmaceutical sectors [171]. However, comprehensive studies evaluating the economic feasibility of these emerging processes in terms of protein recovery remain limited in the literature. Technologies such as supercritical fluid extraction [172] and MAE [173] have demonstrated feasibility for recovering other compounds, such as oils and antioxidants from plant sources. However, these methods, along with other extraction technologies discussed in this article, have not been fully explored from an economic perspective for protein extraction from legumes. This highlights a clear need and opportunity for further research in this area, aimed not only to establish these methods at the laboratory scale but also at advancing their feasibility for industrial application.

### 5.2. Economic Viability: Market Size and Challenge of Plant-Protein Costs

The plant-based protein market size is projected to be valued at USD 19.2 billion in 2028 [174]. Like any source of alternative proteins, investments in legume-protein markets are needed to support the development and commercialization of products at affordable prices. However, most of the investments have been applied to high-income countries [175]. The costs of plant protein were assessed to be approximately 3-fold cheaper than animal protein [176]. However, market prices do not represent accurately the real price of a product. Currently, the costs of commercialized plant-based proteins are approximately 40% higher than animal protein [177].

The increase in market competition aimed at reducing the costs of legumes as an alternative protein source in low- and middle-income countries was hypothesized by Talwar et al. [175]. However, Versa et al. observed that the adoption of sustainable practices, such as environmentally friendly packaging and organic certification, led to higher prices for precooked and dried lentils in the Italian retail market [178]. Rogers et al. reported that high costs of pea protein were attributed to limited processing infrastructure and high-quality pea sourcing. The taste of pea protein limits consumer acceptability, which increases the costs with additional processes for taste-masking [179]. After the taste, price is the second factor that influences the purchase intention of a plant-based product [180]. Varela-Ortega et al. reported that plant protein-based products may be economically attractive via the development of optimized supply chains and processing methodologies [181]. The utilization of waste streams of legume processing has emerged as an alternative to decrease the costs of legume protein-based products. Zhang et al. evaluated the use of pea, chickpea and soybean wastewater as an egg substitute in the preparation of sponge cakes [182]. Plazzotta et al. studied the application of okara as a fat replacer in sweet breads and observed similarities with palm oil breads. Okara is a waste stream of soy milk or tofu processing [183]. The extraction yield and the protein content in the extracted protein influences the cost of the final product and consequently, the economic feasibility of a process. Process feasibility is possible to be assessed after careful selection of raw materials with high protein composition, efficient extraction methodologies (low solvent, energy, and time consumption), the country and local chain supplies, and fees, as well as quality demands.

### 5.3. Environmental Aspects

Sustainability has been extensively demanded in the development of products across the food chain to deliver food security and to reduce climate change. However, the assessment of environmental impacts analyzed via the life cycle assessment of the processes applied to produce legume protein is still under-investigated to the best of the authors’ knowledge.

Currently, the largest contribution to environmental impacts is attributed to food production, from farm to fork [184]. Hueppe and Zander hypothesized that increasing legume consumption and sustainability consciousness of consumers in Germany can improve the food systems in the European Union, which are currently not considered sustainable [185].

Even though plant protein sourcing was demonstrated to produce less environmental impact compared to animal protein [186], the complete green footprint in processing is another challenge faced by the legume-protein industry. For instance, in comparison to a beef burger, the environmental impacts of burger patties formulated with pea protein isolate were lower in terms of climate change, acidification, and eutrophication, but higher in terms of water consumption and human carcinogenic, marine, freshwater, and terrestrial ecotoxicity [184].

The use of renewable energy sources is commonly suggested to decrease the environmental impacts in terms of carbon footprint, ionizing radiation, and ecotoxicity. For instance, Pérez et al. reported that for a small facility that produces white beans in Spain, electricity consumption was the main contributor to their carbon footprint [187]. For the manufacturing of faba bean protein concentrates, Heusala et al. reported that the cultivation conditions (phosphorous fertilizer and fuel consumption) of faba were considered the major contributor to their carbon footprint [188].

### 5.4. Allergenic Potential

The allergenicity of legume proteins is a critical consideration in their current and potential applications as food ingredients. Food allergies are a major health concern, affecting millions worldwide, with approximately 5% of adults and 8% of children impacted [189]. Among allergenic sources, plant-based allergens, including legumes, account for a significant proportion of food allergies. Legumes, widely utilized as protein sources, are particularly noteworthy due to their potential to trigger allergic reactions, affecting 1 in 100 adults and 1 in 10 children [189,190]. As discussed, emerging methods for producing protein isolates and concentrates from legumes such as peas, lentils, lupins, fava beans, and chickpeas have gained attention for their ability to enhance extraction yield and protein functionality. However, many of these studies overlook a crucial aspect, the allergenicity of the final products. This omission is significant, given the high rates of cross-reactivity observed among legumes [190]. Individuals allergic to one type of legume may also exhibit sensitivity to others, though not universally to all types.

Additionally, it is important to note that many protein extraction methods not only aid in protein recovery but also modify the proteins (secondary and tertiary structures). These modifications could potentially alter the immunoreactivity profile of the processed proteins, emphasizing the need for thorough evaluation of allergenic risks during product development. Addressing this biological property is essential to ensure the safety and acceptability of legume-based protein products in food applications. Strategies to mitigate allergenicity include the use of thermal treatments (e.g., boiling or roasting), enzymatic hydrolysis, and other non-conventional processing methods, such as Mw processing or PEF treatments [191,192,193,194]. These techniques can reduce allergenic potential by modifying protein structures, potentially decreasing the protein’s ability to bind to IgE antibodies in sensitized individuals. Pretreatments like soaking or fermentation can also be effective in reducing antigenicity by promoting the breakdown of allergenic proteins. It is crucial that further studies focus on evaluating the effects of these treatments on allergenic proteins and explore optimal combinations for allergen reduction.

### 5.5. Sensory Aspects

The sensory properties of plant-derived proteins remain a major challenge. Consumer acceptance is often low, mainly due to undesirable attributes such as off-flavors, bitterness, or grainy textures, particularly in legume-based ingredients. These perception issues are largely linked to the physicochemical characteristics of the proteins. Rich in polyunsaturated fatty acids (PUFAs) like linoleic and α-linolenic acids, plant proteins often produce fats that are liquid at room temperature. These fats lack the functional properties of saturated fats, which are essential for desirable characteristics such as butter spreadability, whipped cream foaming, and the creamy texture of ice cream [186]. Furthermore, PUFAs are highly prone to oxidation, leading to the formation of volatile compounds that negatively impact sensory attributes, including odor and texture [195]. To address these issues, strategies such as antioxidants, encapsulation, and microgels have been investigated [186]. The oxidative sensitivity of PUFAs also highlights the importance of lipid removal prior to protein extraction. Effective defatting strategies, such as solvent extraction, cold pressing, or supercritical CO_2_ extraction, can significantly reduce lipid oxidation, thereby improving the stability and quality of the final protein product. Legume-based beverages often exhibit bitterness due to antinutritional factors, hydrolyzed peptides, and oxidized lipids, which are difficult to be eliminated [196]. Key antinutritional factors associated with bitterness include saponins, alkaloids, and phenolic compounds [196]. To improve the nutritional quality of legume protein extracts, several techniques can be applied to reduce or eliminate antinutritional factors. Soaking and washing the seeds, particularly when combined with fermentation or thermal treatments, are effective in lowering the levels of saponins, alkaloids, and phenolics, thereby improving flavor and nutritional quality [197,198]. Furthermore, fermentation with bacteria or fungi can be used to degrade antinutrients like phytic acid and tannins, which affect mineral and protein bioavailability as well as sensory properties [199]. Thermal treatments such as boiling or autoclaving are also effective in destroying heat-sensitive antinutrients. In some cases, solvent extraction, using ethanol or methanol, is employed to remove bitter or toxic compounds from plant sources such as chlorogenic acid [95]. Enzymatic treatments with proteases can further enhance flavor by breaking down antinutritional proteins and minimizing the formation of bitter peptides [200]. These treatments improve the palatability and increase the bioavailability of nutrients, making legume protein extracts more suitable for food applications. For instance, soymilk, although widely consumed, continues to suffer from beany flavor, off-notes, and poor stability [186]. Some studies have evaluated the sensory performance of legume proteins in specific applications. For example, bread with lupin protein isolates (up to 9%) or concentrates (up to 3%) showed no sensory differences compared to bread made entirely with wheat flour [201]. Similarly, partially substituting millet flour with 10% pea protein, combined with transglutaminase, enhanced structure, specific volume, softness, and sensory quality while reducing millet’s bitterness [202]. These findings highlight the potential of plant-based proteins to replicate desirable sensory and structural qualities in food products, addressing consumer demand for alternatives that retain the texture, mouthfeel, and satiety traditionally associated with animal-based foods. However, further research is still needed to fully understand and optimize the organoleptic properties of plant-derived products, taking into account the diversity of available sources and their specific applications, such as meat and dairy analogs.

### 5.6. Synergistic Potential of Legume Proteins with Other Protein Sources

The combination of legume proteins with other protein sources, such as microbial (bacterial, fungal, and algal), animal proteins, or other plant protein sources represents a promising strategy to enhance the functional and nutritional properties of plant-based ingredients [203]. Mixing different proteins with complementary amino acid compositions enhances the nutritional quality of food. For instance, legume proteins are often deficient in sulfur-containing amino acids [204], which can be balanced by combining them with fungal or animal proteins. Furthermore, other plant-based proteins, such as those derived from cereals, seeds, and nuts, can also complement legume proteins. These combinations can improve the amino acid profile and nutritional quality of the final product. A review by Jiménez-Munoz et al., 2021 [203], highlighted that mixing different protein sources (e.g., cereals, algal) can improve techno-functional properties, such as solubility, emulsification, gelation, and digestibility, all of which are essential for applications in meat substitutes, dairy analogs, and other structured food products. Furthermore, hybrid protein formulations can contribute to both sustainability and innovation in food design. The incorporation of microbial proteins from algae or mycelium, for instance, can boost protein yields with a lower environmental footprint, while also offering unique textures and flavors. Combining legume proteins with other protein sources in hybrid products also provides a potential pathway for consumers transitioning to plant-based diets, delivering enhanced sensory characteristics while decreasing dependence on traditional animal agriculture [205]. Exploring these synergies may lead to the development of innovative food formulations that meet consumer expectations and align with sustainability objectives.

## 6. Conclusions and Future Perspectives

Legume proteins are gaining significant attention as an alternative to animal-based proteins due to their nutritional value, functional properties, and potential benefits for plant-based food formulations. However, challenges persist, particularly in terms of optimizing extraction methods, improving protein functionality, enhancing consumer sensory acceptance, and addressing the allergenic potential of certain legume proteins. Despite advancements in emerging extraction technologies such as UAE, MAE, and OH, their application to legume proteins remains under-researched, and their scalability for industrial production is not fully established.

In the near future, further investigation is required to explore the economic feasibility and environmental impact of these novel extraction techniques. Additionally, studies are needed to assess the impact of various extraction conditions on protein functionality and digestibility. The role of specific legume varieties in protein yield and quality should also be explored. Furthermore, addressing the allergenic potential of legume proteins, through modification or purification techniques, will be crucial to enhance their safety profile for broader consumer acceptance. Future perspectives also involve the integration of waste valorization strategies, where by-products from legume processing could be repurposed into valuable ingredients, contributing to the development of a circular economy. Establishing sustainable and cost-efficient processing technologies, alongside a well-organized supply chain, will be key in overcoming the challenges of scaling up legume protein production for commercial use. As the demand for plant-based proteins continues to rise, ongoing research into optimizing extraction methods and improving protein characteristics will be pivotal in making legume proteins a viable, competitive option in the global protein market.

## Figures and Tables

**Table 1:** Traditional and non-traditional legume sources.

Sources	Protein Content (%)	Reference
Soybean	35–40	[36]
Pea	23–27	[37]
Beans:		
Faba bean	20–41	[38]
Mung bean	21–31	[39]
Green bean		[40]
Black bean	20–30	[41]
Azuki bean	19.90	[40]
Cranberry bean	23.00	[40]
Ganxet bean	24–29	[42]
Kidney bean	22.53	[40]
Jack bean	23–35	[22]
Pigeon pea	18–28	[21]
Lentil	22–30	[41]
Hyacinth bean	22–25	[26]
Chickpea	20–24	[40,43]
Lupins	29–55	[7]
Acacia	18–36	[32]
Peanut	26–29	[44]
Cowpea	23–32	[45]

## Data Availability

Not applicable.
